# Signatures of new *d*-wave vortex physics in overdoped Tl_2_Ba_2_CuO_6+*x*_ revealed by TF-*µ*^+^SR

**DOI:** 10.1038/srep14156

**Published:** 2015-09-16

**Authors:** Jess H. Brewer, Scott L. Stubbs, Ruixing Liang, D. A. Bonn, W. N. Hardy, J. E. Sonier, W. Andrew MacFarlane, Darren C. Peets

**Affiliations:** 1 Department of Physics and Astronomy, The University of British Columbia, Vancouver, BC, Canada V6T 1Z1; 2 Canadian Institute for Advanced Research, Toronto, Ontario, Canada M5G 1Z8; 3 Department of Physics, Simon Fraser University, Burnaby, BC, Canada V5A 1S6; 4 Department of Chemistry, The University of British Columbia, Vancouver, BC, Canada V6T 1Z1; 5 Center for Correlated Electron Systems, Institute for Basic Science, Seoul National University, Seoul 151-747, Korea

## Abstract

The spontaneous expulsion of applied magnetic field, the Meissner effect, is a defining feature of superconductors; in Type-II superconductors above the lower critical field, this screening takes the form of a lattice of magnetic flux vortices. Using implanted spin-1/2 positive muons, one can measure the vortex lattice field distribution through the spin precession and deduce key parameters of the superconducting ground state, and thereby fundamental properties of the superconducting pairing. Muon spin rotation/relaxation (*µ*SR) experiments have indeed revealed much interesting physics in the underdoped cuprates, where superconductivity is closely related to, or coexistent with, disordered or fluctuating magnetic and charge excitations. Such complications should be absent in overdoped cuprates, which are believed to exhibit conventional Fermi liquid behaviour. These first transverse field (TF)-*µ*^+^SR experiments on heavily-overdoped single crystals reveal a superfluid density exhibiting a clear inflection point near 0.5*T*_c_ , with a striking doping-independent scaling. This reflects hitherto unrecognized physics intrinsic to *d*-wave vortices, evidently generic to the cuprates, and may offer fundamentally new insights into their still-mysterious superconductivity.

Charge doping of the CuO_2_ planes tunes the occurrence of superconductivity in the high-temperature hole-doped cuprate superconductors between the limits of an undoped insulating antiferromagnet and a possible conventional Fermi liquid at high dopings. It is appealing to try to understand how the unconventional superconductivity evolves out of these more conventional electronic ground states. However, hole doping is typically effected chemically, in the best case *via* the composition of a distinct, well-separated subunit of the layered crystal structure, to leave the planes themselves little altered structurally and the dopant site well-shielded when a hole is promoted to the planes. One such example is oxygen doping in the CuO chain layer of YBa_2_Cu_3_O_7−*δ*_ (YBCO). Unfortunately, compositional tuning is limited by (thermodynamic) phase stability and can seldom be used to traverse the entire superconducting phase diagram in a single system. In this context overdoped cuprates, those nearer the apparent Fermi liquid regime, are rare and, moreover, relatively few have highly-ordered CuO_2_ planes. For example, doping by cation substitution in La_*x*_Sr_2−*x*_CuO_4_ (LSCO) introduces substantial disorder directly adjacent to the CuO_2_ planes^1^. In contrast, Tl_2_Ba_2_CuO_6+*x*_ (Tl-2201) offers tunability throughout the overdoped regime with highly-ordered, isolated, and flat CuO_2_ planes, doped *via* dilute interstitial oxygen in the distant TlO layers^2^, although Cu/Tl substitution in this layer^3^,^4^ may contribute an offset in doping. The overdoping also appears to eliminate a predicted electron Fermi surface (FS) pocket at the Γ point, leaving only a single, large, FS sheet^5^.

Pure 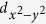-wave symmetry of the superconducting order in Tl-2201 has been conclusively established by observation of half-integer flux quanta at crystal boundaries in films^6^; line nodes are evident in microwave^7^,^8^,^9^ and thermal transport^10^ measurements; and the admixture of another pairing symmetry is unlikely because it would require spontaneous breaking of the crystal symmetry. However, some *μ*SR measurements have suggested an additionial transition at low temperatures within the vortex state^11^,^12^,^13^, which has been interpreted in terms of a multiple-component order parameter. Here, we extend *μ*SR studies of the vortex state of the cuprates deep into the overdoped regime with the first transverse-field muon spin rotation (TF-*μ*^+^SR) results on single-crystalline Tl-2201, in the form of high-quality single crystal mosaics at a range of dopings. Our measurements were performed at low magnetic fields, in a doping regime free from competing charge density wave order^14^,^15^ and are thus sensitive to the intrinsic structure of *d*-wave vortices. We show that the unusual temperature dependence is real, and generic to the cuprates, but also demonstrate that it is a signature of *d*-wave vortex physics rather than a multicomponent order parameter.

## Results

**Figure 1:**
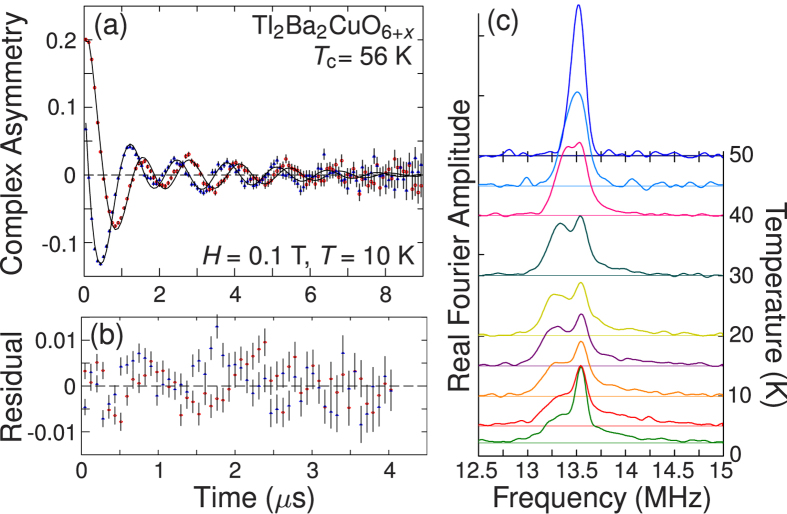
Example of *μ*^+^SR data. (**a**) Complex TF-*μ*^+^SR time spectrum (red circles: real part; blue triangles: imaginary part) in a rotating reference frame (RRF) at 0.1 T and 10 K on the 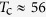 K Tl-2201 mosaic, including time-domain best fit, the residual errors of which are shown in (**b**) for the first 4 *μ*s where the statistics are highest. (**c**) Fourier transforms at several temperatures. The relatively sharp peak at 13.55 MHz arises from muons stopping outside the sample.

Figure 1 shows an example of the data and time domain fit at 10 K on a *T*_c_ = 56 K mosaic. Fits on all mosaics at all fields and temperatures converged very well, and fully reproduce the data. Fourier transforms corresponding to the field distribution are also shown for a selection of temperatures; the additional peak just above the cusp is attributed to muons stopping outside the sample and precessing about the applied field, and is accounted for in the fits. The high-field (high-frequency) cutoff in the lineshape is indistinct, precluding a quantitative analysis of the in-plane coherence length 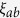, but the in-plane magnetic penetration depth 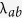, which controls the linewidth, may be reliably extracted. Varying the fit parameters indicated that the absolute 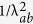 is accurate to within ~10%, while its temperature dependence is robust; after a few global fits with different choices of 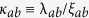 we chose a fixed value, 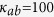, for all remaining fits.

One strength of TF-*μ*^+^*SR* in a Type-II superconductor is its ability to determine the *absolute* λ and its inverse square, which is proportional to the density of superconducting carriers^16^. Circumstances are not yet as good for Tl_2_Ba_2_CuO_6+*x*_ as for high-quality YBCO; in particular, only small improvements of global *χ*^2^ minimization distinguish the broadening due to vortex lattice disorder, *σ*_*d*_, which should scale with 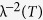, from *T*-independent broadening due to nuclear magnetic dipoles and crystal defects, *σ*_0_. The amplitude *A*_*B*_ of the background signal due to muons stopping outside the sample is also known only from the best fit; like *σ*_0_, it can be subtly coupled to λ^−2^. These uncertainties do not alter the temperature dependence, and have been incorporated into the quoted ~10% uncertainty in 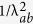.

**Figure 2:**
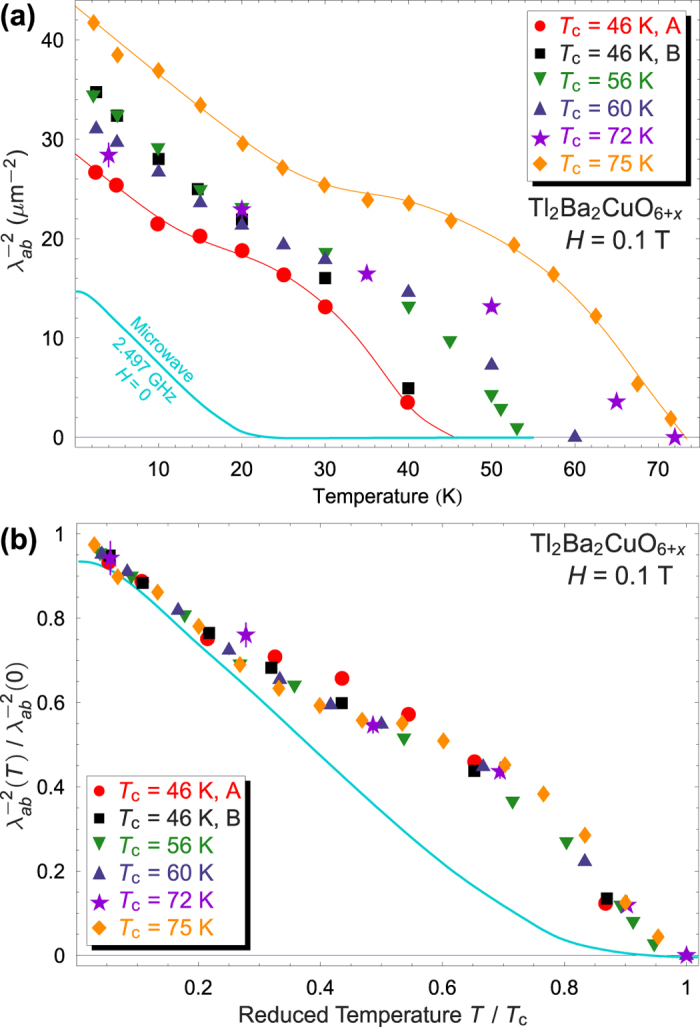
(**a**) Temperature dependence of fitted 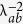 at *H* = 0.1 T for all Tl-2201 mosaics; A and B denote two different mosaics with the same *T*_c_. Absolute microwave data (curve) at zero field on a *T*_c_ = 25 K crystal at 2.497 GHz^9^, included for comparison, follow a qualitatively different form. Curves are provided for two mosaics as a guide to the eye. (**b**) Normalized values 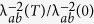 *vs*. reduced temperature *T*/*T*_c_ for all Tl-2201 mosaics. All dopings exhibit essentially the same temperature dependence, and differ from the microwave results (solid curve).

Figure 2 shows the vortex-state 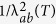, which is proportional to the superconducting carrier density, for the six mosaics measured, and Table 1 reports the zero-temperature penetration depth 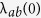 from linear extrapolations of 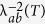. A highly unusual *T*-dependence, common to all dopings, is immediately apparent. The extent of this similarity is more striking when 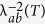 is normalized to its extrapolated *T* = 0 value and plotted against reduced temperature *T*/*T*_c_—the *relative* temperature dependence is *identical*. The most intriguing feature, exhibited in all six mosaics, is upward curvature between 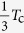 and an inflection point around 0.5*T*_c_. This unusual temperature dependence is robust and evident in any measure of the linewidth, but is absent in zero-field (Meissner state) microwave surface resistance at higher and lower dopings^8^,^9^, the former included for comparison. The intrinsic *T*-dependence of the superconducting carrier density (or 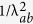) in a single-gap *s*- or *d*-wave superconductor exhibits downward curvature over the entire temperature range 0–*T*_c_.

## Discussion

*μ*SR reports of the cuprates’ temperature-dependent in-plane penetration depth typically exhibit the shape associated with a pure *d*-wave order parameter^17^,^18^. However, this has not been the case in all data. Upward curvature in the *μ*SR penetration depth can be recognized in relatively disordered overdoped LSCO^11^; at high dopings in cleaner YBa_2_Cu_3_O_7−*δ*_^19^, in lightly underdoped YBa_2_Cu_4_O_8_^12^; and in optimally and overdoped Bi_2_Sr_2_CaCu_2_O_8+*δ*_^13^. This unusual temperature dependence has appeared most clearly near and above optimal doping^18^,^19^, with some limited evidence that it strengthens on overdoping^13^. It is most evident at relatively low applied fields^20^,^21^.

With no phase transition expected at the fields and temperatures in question, no such feature unambiguously visible in most published data or any microwave penetration depth studies, and based on a small number of data points in many of these cases, it has not been widely accepted as a real effect. Its now-confirmed appearance in a variety of systems, and its particularly conspicuous appearance in Tl-2201, implies that it is real and generic, at least to high doping ranges. The contrast with zero-field microwave data argues against the few interpretations floated thus far, instead pointing toward an origin in an unexpected property of the cuprates’ vortex state. We first briefly dispense with some alternative explanations before returning to vortex physics.

First, multiband superconductors, those with more than one band crossing the Fermi level, can exhibit unconventional temperature dependence in the vortex state^22^; a two-component order parameter, *e.g. d* + *s*, with separate order parameters on distinct Fermi surface sheets, has been advanced to explain the LSCO^11^ and YBa_2_Cu_4_O_8_^12^ results. However, Tl-2201 has only one FS sheet, and the pure *d*-wave symmetry and dissimilar microwave penetration depth at both lower and higher dopings^7^,^9^ exclude such an origin. Second, the remarkable scaling seen in Fig. 2 argues against an electronic phase transition within the superconducting dome, such as an extension of the pseudogap crossover temperature T^*^. Third, dilute paramagnetic impurities would yield an additional broadening scaling with *H*/*T*, contrary to the observed *T* dependence. Some type of magnetically frozen state might account for the observed temperature dependence (since *μ*^+^SR is a local probe, macroscopic phase separation would not affect the superconducting component’s lineshape). However, one would not expect the onset of a competing magnetic phase to track *T*_c_ with doping^23^. Furthermore, higher fields should enhance any competing magnetic order^24^, particularly in the vortex cores^25^, but in YBCO they instead suppress the exotic upward curvature in the temperature dependence of the linewidth^11^,^20^. Proximity-induced chain superconductivity has been advanced as an explanation for the inflection point in YBCO^19^, but this cannot explain its appearance in chain-free Tl-2201.

Having excluded several alternative explanations, we return to physics of the vortex phase, which would be absent in the Meissner-phase microwave experiments and previous work on Tl-2201 powder^26^, and may thus offer a natural explanation. First, the resistive upper critical field of Tl-2201 (actually the irreversibility field^27^,^28^) exhibits unusual upward curvature^29^ and stays very close to *H*_c2_(*T*), far from the required temperature regime at the low fields relevant here. A dimensionality crossover within the frozen vortex state, as in the much more anisotropic Bi_2_Sr_2_CaCu_2_O_8_^13^,^30^, would produce a symmetric lineshape, and the reduced field inhomogeneity would narrow the linewidth at high temperatures, in contrast to our results. Trapping of vortices by preferred pinning sites at low temperature has been advanced to explain the inflection in YBCO^20^, but the linewidth changes in Tl-2201 would require *very* significant disorder and change the lineshape significantly. This is not seen in the FFT spectra; moreover, allowing the temperature-independent *σ*_0_ broadening to vary produced no systematic trend with temperature. To further exclude vortex disorder, it will be essential to quantify it independently using, for instance, small-angle neutron scattering or scanning probe techniques.

Another relevant feature of the vortex state is the symmetry of the vortex lattice itself. An unusual square vortex lattice, as found in optimally and overdoped LSCO^31^,^32^ and in YBCO^33^, produces a broader but qualitatively similar field distribution with a more pronounced low-field tail. However, the vortex lattice in fully-oxygenated YBCO gradually transforms from triangular to square as the field increases from 4 to 11 T, while strong upward curvature in the second moment of the TF-*μ*^+^SR lineshape is strongest at much lower fields ~0.1 T^34^ and is completely suppressed by 4 T^18^, excluding this interpretation, at least for YBCO. The *μ*SR data for the *T*_c_ = 56 K Tl-2201 mosaic were fit to a simple square vortex lattice model as a trial, but no crossover, in the form of a systematic improvement in the quality of fit, was evident.

We are thus drawn to the conclusion that the upward curvature must arise from some fundamental property intrinsic to the *d*-wave vortices themselves. In this scenario, the difference between the zero-field microwave measurements and these vortex-state *μ*SR results arises because the measurements probe different phases. In microwave measurements, only the surface contributes, while the bulk is shielded. In *μ*SR, however, vortices also contribute, and these can overlap and interact in certain field and temperature regimes.

The scaling with *T*_c_ and absence in underdoped samples point to an explanation in terms of the electronic structure expected for a vortex in a *d*-wave superconductor. Theoretical calculations^35^,^36^ show that such vortex cores shrink with increasing magnetic field due to an enhanced transfer of quasiparticles between neighbouring vortices. This effect has been observed in YBCO by *μ*SR^18^,^19^,^37^, with the core size rapidly shrinking and saturating at fields above *H* ~ 4 T. At low fields where the vortices are well separated, the quasiparticle transfer is minimal and the vortices are expected to behave essentially as if they were isolated. A vortex in a *d*-wave superconductor is fourfold symmetric at low temperatures, with extended low-energy quasiparticle states along the 45° (nodal) directions in the CuO_2_ plane. With increasing thermal population of the higher energy states, the vortex core size grows, and calculations show that above ~0.5*T*_c_ the fourfold-symmetric magnetic field profile about the vortex core becomes nearly cylindrical^38^.

As previously stressed^25^, λ_*ab*_ as measured by *μ*SR may be regarded as the in-plane magnetic penetration depth only in the 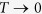 and 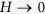 limit, but is otherwise an effective length scale partially influenced by changes to the field profile outside the vortex core by extended quasiparticle states. Consequently, below ~0.5*T*_c_ and at low fields the temperature dependence of λ_*ab*_ is expected to be influenced by the evolving quasiparticle states that extend far beyond the vortex core. At higher temperatures, where the fourfold symmetry of the vortex is essentially gone, the behavior of λ_*ab*_ should closely resemble that of the magnetic penetration depth, unless the vortex lattice melts. Since the vortex core radius is directly proportional to the gap magnitude^39^, which in turn is proportional to *T*_c_ in overdoped cuprates, the scaling with *T*_c_ is naturally explained.

The inflection point near 0.5*T*_c_ being less prominent or absent in underdoped cuprates is likely due to the stabilization of competing charge-density-wave (CDW) order localized in the vicinity of the vortex cores. STM measurements on optimally- and slightly overdoped Bi_2_Sr_2_CaCu_2_O_8+*δ*_^40^,^41^ and nuclear magnetic resonance (NMR) measurements on underdoped YBCO^24^,^42^ show static CDW order in the vortex core region, where superconductivity is suppressed. While this induced static CDW order is observed only at low temperatures in optimally and slightly overdoped samples, in underdoped YBCO with *p* ~ 0.12, NMR shows that static CDW order occurs over much of the temperature range below *T*_c_. The occurrence of static CDW order in the vortex core region constitutes a significant modification of the electronic structure of the *d*-wave vortex^43^, and consequently the loss of the inflection point in the temperature dependence of λ_*ab*_ is not surprising. The CDW competes with superconductivity^14^,^15^, so it will preferentially inhabit—and likely gap out—the regions of momentum space in the nodal direction at the Fermi surface to maximally avoid competition between the two orders. As a result, the extended quasiparticle core states along the nodal directions will become bound. This should lead to isotropic *s*-wave-like vortex behaviour throughout much of the underdoped side of the phase diagram, while the behaviour seen in the overdoped regime reflects the intrinsic physics of *d*-wave vortices without such complications. Supplementary Fig. S2 online shows that the fit parameters, based on an *s*-wave model, fail at the lowest temperatures (beginning well below the inflection point). The *s*-wave model’s inability to reproduce the observed field distribution provides evidence of its failure to adequately describe the vortex phase, particularly at low temperatures.

**Figure 3:**
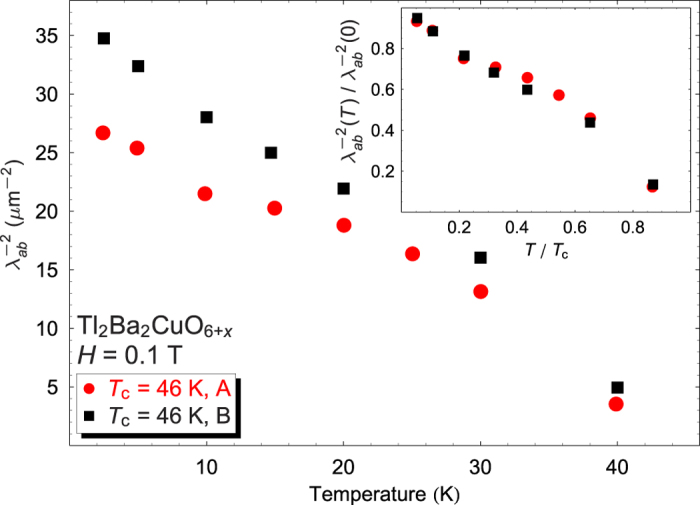
Fitted values of 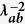 *vs.T* for the earlier (A) and later (B) *T*_c_ = 46 K mosaics. Inset: same data in normalized form.

Finally, our use of mosaics with similar *T*_c_s has important implications for techniques relying on *μ*SR for values of the zero-temperature penetration depth. Mosaics grown and annealed under very similar conditions, having the same or similar *T*_c_, exhibit quite different absolute penetration depths, as shown in Fig. 3 for *T*_c_ = 46 K. The *normalized* linewidths 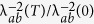, however, are almost identical. Mosaics with *T*_c_s of 46 K (“A”) and 72 K were prepared several years before the 46 K (“B”) and 75 K mosaics. The crystal growth was still being optimized when the early mosaics were assembled, demonstrating that suppression of *T*_c_ by disorder is *not equivalent* to its suppression by carrier overdoping. That the zero-temperature 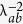 can apparently increase by ~30% due to increasing crystalline perfection means that the λ_*ab*_(0) values in Table 1 should *not* be regarded as intrinsic. A variety of other techniques rely upon *μ*SR to obtain absolute penetration depth values, but our work indicates that in the overdoped regime, the values are only valid for 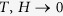, and even then are highly susceptible to disorder and must be treated with caution.

The nature of superconductivity in the cuprates remains one of the most important open questions in condensed matter physics, and the overdoped regime proffers the promising prospect of understanding the normal state from which high-temperature superconductivity emerges. However, our *μ*^+^SR data indicate exotic physics survives to high dopings. A striking universal temperature dependence unambiguously confirms an unusual upturn in the in-plane penetration depth, now seen in at least four distinct material families, establishing that it is new physics generic to the cuprates. Fundamental differences from zero-field microwave measurements of nominally the same quantity^9^ imply it is intrinsic to the *d*-wave vortices themselves. Aside from the substantial impacts of this result on vortex physics and what light this may shed on cuprate superconductivity, there are important ramifications for other techniques. *μ*SR is uniquely suited to extracting the absolute penetration depth from the vortex phase, and the values thus obtained underpin results of other techniques which can’t measure λ in absolute terms or at all. While the *μ*SR temperature evolution should be robust^16^, the absolute values are model-dependent. Our data indicate that a more complex model is required when *d*-wave vortices are present, which will necessitate revisiting some previous results. First, however, the details and origin of the exotic temperature dependence must be conclusively confirmed. Sensitive scanning probe microscopies may provide insights into the current and quasiparticle distribution in the vortex cores, while confirmation that this is a vortex state phenomenon awaits Meissner-state field distribution measurements, such as low-energy *μ*SR^44^ or *β*-NMR^45^. The regimes in which these *d*-wave vortices manifest their unique *d*-wave physics may provide crucial new insight on the cuprates’ order parameter and still-mysterious pairing.

## Methods

Single crystals of Tl-2201 were grown in gold-sealed alumina crucibles by an encapsulated copper-rich self-flux method as described elsewhere^46^. The oxygen content (which determines hole content and *T*_c_) was set by annealing under controlled oxygen partial pressures and temperatures^47^; two different annealing schemes were employed depending on the desired oxygen content^46^. Crystals were assembled in mosaics on substrates of aluminized mylar or GaAs to minimize the background signal, with the crystallographic *c*-axis perpendicular to the substrate (parallel to the applied field). In this geometry, the applied field is shielded by supercurrents running within the *ab*-plane, thus the in-plane penetration depth λ_*ab*_ governs the field distribution. The large number of small crystals to be mutually aligned precluded measuring the superconducting transition of every individual crystal, but care was taken to construct each mosaic from a small number of annealing batches, each drawing crystals from only one growth run, and *T*_c_ was measured on a selection of crystals sampled from each annealing run. Examples of magnetization data on three mosaics are included in Supplementary Fig. S1 online. Quoted uncertainties in *T*_c_ reflect transition widths of individual crystals, as determined by DC magnetization in applied fields of 0.1–0.2 mT, and the expected variation within the mosaic based on the crystals sampled. These fields were used because the lower critical field *H*_c1_ in this material is quite low.

Spin-polarized positive muons from the M15 muon channel at TRIUMF were injected into the mosaics at an energy of 3–4 MeV in one of several different *μ*^+^SR spectrometers. While a range of magnetic fields were used, the lowest field for which the rotating reference frame transformation worked reliably was 0.1 T, and this field was used for all data presented here. In muon spin rotation/resonance/relaxation (*μ*SR)^16^,^48^, implanted muons settle into specific preferred crystallographic sites, where their spins precess around the local magnetic field 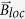 with frequency 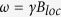, where the muon gyromagnetic ratio 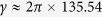 MHz/T. The precession is detected *via* a decay positron (in the case of *μ*^+^) emitted preferentially along the muon’s spin direction. The experimental *β*^+^ decay asymmetry reflects the precession of the ensemble of ~10^7^ randomly implanted muons and thereby the distribution of the local magnetic field. A Fourier transform of the time spectrum, which can be useful for visualizing the field distribution characteristic of the vortex state, shows a low-field cutoff corresponding to the midpoint of a triad of near neighbor vortices, a van Hove cusp from saddle points in the vortex lattice, and a high-field tail and cutoff corresponding to the maximum *B*_*loc*_ in the vortex cores. However, fitting is best performed on the original time spectra: statistics decay with the muon lifetime (2.197 *μ*s), and mixing high- with low-statistics data (as in an FFT) is undesirable, but later times determine the frequency resolution and thereby the reliability of fit parameters. All λ_*ab*_ values were therefore extracted from fits in the time domain to the lineshape described in Ref. 49 as calculated numerically for a triangular vortex lattice with λ_*ab*_ and 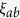 as fitted parameters. A test with a square vortex lattice used the same approach.

## Additional Information

**How to cite this article**: Brewer, J. H. *et al.* Signatures of new *d*-wave vortex physics in overdoped Tl_2_Ba_2_CuO_6+x_ revealed by TF-*µ*^+^SR. *Sci. Rep.* **5**, 14156; doi: 10.1038/srep14156 (2015).

## Supplementary Material

Supplementary Information

## Figures and Tables

**Table 1:** Zero-temperature in-plane magnetic penetration depths in 0.1 T for overdoped Tl-2201 mosaics having various *T*_c_s, from a linear extrapolation of 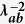
(*T*) at low temperatures, with estimated uncertainties in parentheses.

*T*_c_ (K)	46(1), A	46(1), B	56(1)	60(1)	72(1)	75(1)
λ_*ab*_(0) (nm)	187(2)	165(2)	166(1)	175(1)	182(2)	153(2)

The variations in λ_*ab*_(0) are most likely dominated by the degree of order in the samples, rather than any systematic doping dependence, as discussed in the text. Uncertainties in *T*_c_ represent primarily the variation in *T*_c_ among the crystals comprising the mosaic.
