# Supplementary material for: Signatures of new d-wave vortex physics in overdoped Tl2Ba2CuO6+x revealed by TF-µ+SR
Source: Sci Rep. 2015 Sep 16;5:14156. doi: 10.1038/srep14156 (PMC4570984; doi:10.1038/srep14156)
Supplement: Supplementary Information [file srep14156-s1.pdf]

Signatures of new d-wave vortex physics in overdoped $Tl_2Ba_2CuO_{6+x}$ revealed by TF-μ^+SR: Supplementary Information

Jess H. Brewer,[1,*] Scott L. Stubbs,[1] Ruixing Liang,[1,2] D. A. Bonn,[1,2] W. N. Hardy,[1,2] J. E. Sonier,[3,2] W. Andrew MacFarlane,[4] and Darren C. Peets[1,5,†]

[1] *Department of Physics and Astronomy, The University of British Columbia, Vancouver, BC, Canada V6T 1Z1*
[2] *Canadian Institute for Advanced Research, Toronto, Ontario, Canada M5G 1Z8*
[3] *Department of Physics, Simon Fraser University, Burnaby, BC, Canada V5A 1S6*
[4] *Department of Chemistry, The University of British Columbia, Vancouver, BC, Canada V6T 1Z1*
[5] *Center for Correlated Electron Systems, Institute for Basic Science, Seoul National University, Seoul 151-747, Korea*

The field-cooled magnetization data collected for three mosaics — 46 K (B), 56 K, and 75 K — are shown in Fig. S1. Data were taken on cooling in applied fields of 0.1–0.2 mT to minimize broadening due to the lower critical field. To aid comparison, the data have been normalized. All data were collected with the field applied along the c-axis.

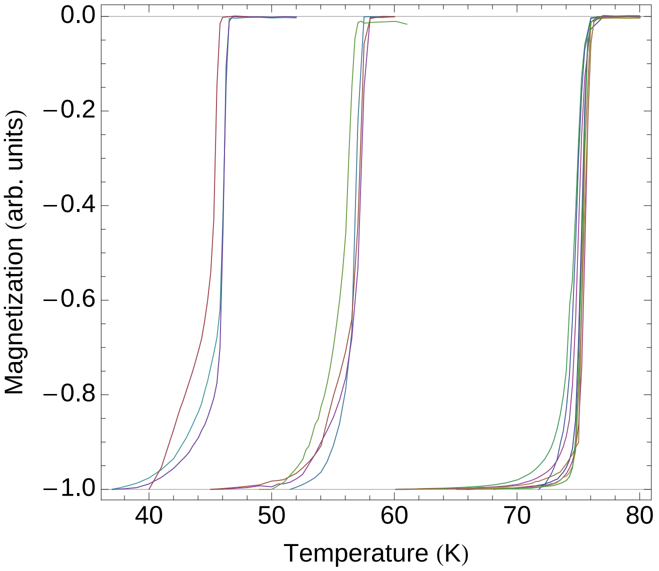

FIG. S1. Example field-cooled magnetization data for three of the mosaics measured in this study.

Figure S2 shows anomalous behaviour in the extracted vortex lattice lineshape, using data collected on the $T_c = 75$ K mosaic at $H = 0.1$ T as a representative example. Because the field is applied by a driven superconducting magnet and can drift by up to 0.5% over the course of a full temperature sweep, frequency values here are corrected by subtracting the frequencies corresponding to the actual applied fields as measured by a Hall sensor. The background peak only drifts significantly near T_c, where it becomes difficult to distinguish from the superconducting signal. The mean frequency (first moment) of the fitted superconducting lineshape drifts higher at low temperature, while the cusp corresponding to saddle points between vortices departs from the background

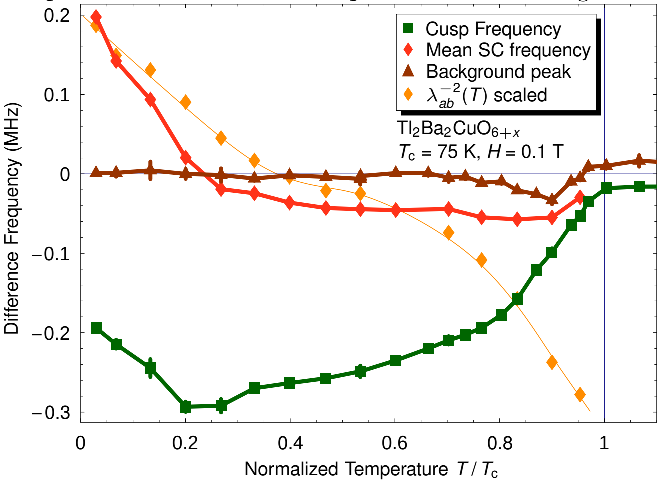

FIG. S2. Anomalous temperature-dependence of fit parameters at $H = 0.1$ T for the $T_c = 75$ K mosaic. The frequency of the background peak, superconducting cusp, and the mean frequency (first moment) of the fitted vortex lattice lineshape are shown, with the frequency corresponding to the applied field subtracted. Rescaled $\lambda_{ab}^{-2}(T)$ values from Fig. 2 are included for reference, again with a curve to serve as a guide to the eye.

on cooling as expected before coming back toward it. In both cases, the departure from expected behaviour occurs at temperatures well below the inflection point in the extracted $\lambda_{ab}^{-2}(T)$. Other fit parameters exhibited no systematic temperature dependence. The anomaly reflects a subtle change in shape of the superconducting field distribution, indicating the breakdown at low temperatures of the s-wave vortex model used, and may provide guidance as to the temperature regime in which d-wave vortex physics must be taken into account.

Raw data for all μSR measurements performed at TRIUMF are freely available at `http://musr.physics.ubc.ca/mud`. Data used in this study may be found by searching for Experiment 958. The current version of LSHfit, used to analyse the data, is available from Jeff Sonier on request.
